# The complete chloroplast genome of *Zoysia matrella* (L.) Merr. isolated in Korea (Poaceae): investigation of intraspecific variations on chloroplast genomes

**DOI:** 10.1080/23802359.2021.1875907

**Published:** 2021-02-12

**Authors:** Bumkyu Lee, Jongsun Park

**Affiliations:** a Department of Environmental Science & Biotechnology, Medicical Science, Jeonju University, Jeonju, Republic of Korea; b InfoBoss Inc., Seoul, Republic of Korea; c InfoBoss Research Center, Seoul, Republic of Korea

**Keywords:** *Zoysia matrella*, Zoysiagrass, chloroplast genome, poaceae, intraspecific variations

## Abstract

Completed chloroplast genome of *Zoysia matrella* (L.) Merr. isolated in Korea is 135,888 bp long (GC ratio is 38.4%) and has four subregions: 81,370 bp of large single copy (36.3%) and 12,594 bp of small single copy (32.7%) regions are separated by 20,962 bp of inverted repeat (44.1%) regions including 130 genes (83 protein-coding genes, eight rRNAs, 38 tRNAs, and one pseudogene). 28 SNPs and 57 INDELs were identified ss intraspecific variations against previously sequenced chloroplast genome. Phylogenetic trees show that *Z. matrella* and *Z. tenuifolia* (=*Z. pacifica*) are clustered in one clade with low level of variations on chloroplast genomes.

Zoysiagrass is an herbaceous and perennial plant and has a wide range of uses such as soil conservation, landscaping, playgrounds, and parks (Bae et al. 2008), and the demand and commercialization are rapidly increasing (Choi J-S et al. 2017). *Zoysia matrella* (L.) Merr is one of the native *Zoysia* spp. in Korea and has a delicate and dense foliage. Molecular breeding has recently been applied to develop new varieties of turfgrass (Bae et al. 2008; Choi J-S et al. 2017). Chloroplast genome of cultivar Chiba Fair Green was sequenced (Tanaka, Hirakawa, et al. 2016) to clarify species identification problem of *Z. matrella* (Tanaka, Tokunaga, et al. 2016) and to unravel their evolutionary relationships. To investigates intraspecific relationship of *Z. matrella*, we completed the chloroplast genome of *Z. matrella* isolated in Korea.

Total DNA of *Z. matrella* isolated from maintained line in National Institute of Agricultural Sciences, Jeonju city, Korea (35°49′47.94ʺN 127°03′55.2ʺE) for conserving natural isolate in Jejudo island in Korea, was extracted from fresh leaves by using a DNeasy Plant Mini Kit (QIAGEN, Hilden, Germany). Voucher was deposited in InfoBoss Cyber Herbarium (IN; http://herbarium.infoboss.co.kr/; Suhyeon Park; shpark817@infoboss.co.kr) under the voucher number IB-01031. Genome was sequenced using NovaSeq6000 at Macrogen Inc., Korea, and *de novo* assembly and confirmation were performed by Velvet v1.2.10 (Zerbino and Birney 2008), SOAPGapCloser v1.12 (Zhao et al. 2011), BWA v0.7.17 (Li 2013), and SAMtools v1.9 (Li et al. 2009) under the environment of Genome Information System (GeIS; http://geis.infoboss.co.kr/; Park et al., in preparation). Geneious R11 v11.0.5 (Biomatters Ltd., Auckland, New Zealand) was used for annotation based on *Z. matrella* chloroplast (AP014937; Tanaka, Hirakawa, et al. 2016).

Chloroplast genome of *Z. matrella* (GenBank accession is MT983887) is 135,888 bp (GC ratio is 38.4%) and has four subregions: 81,370 bp of large single copy (LSC; 36.3%) and 12,594 bp of small single copy (SSC; 32.7%) regions are separated by 20,962 bp of inverted repeat (IR; 44.1%). It contains 130 genes (83 protein-coding genes, eight rRNAs, 38 tRNAs and one pseudogene); 19 genes (seven protein-coding gene, four rRNAs, and eight tRNAs) are duplicated in IR regions. Interestingly, *trnfM-CAU* pseudogene was found at 37,280 to 37,333, where hotspot of pseudogenes identified in *Triticum aestivum* chloroplast genome (Ogihara et al. 2002)

28 SNPs and 57 INDELs were identified from the pair-wide alignment of two *Z. matrella* chloroplast genomes. 8 out of 57 INDELs are 2-bp long and three 3-bp INDELs, two 4-bp INDELs, one 5-bp, and one 10-bp INDEL are also identified. Numbers of SNPs and INDELs are less than those of species between two countries (Jeon et al. 2019; Oh and Park 2020; Park, Kim, Lee 2019); however, similar to those identified inside Korea (Choi YG et al. 2019; Kim, Yi, et al. 2019) and even larger than several plants *(*Park and Kim 2019; Kim et al. 2020; Min et al. 2019; Park and Oh 2020; Park, Kim, Xi, et al. 2019), indicating that number of intraspecific variations of *Z. matrella* between Korea and Japan is relatively small.

Eleven chloroplast genomes including two *Z. matrella* chloroplasts were selected from phylogenetically neighbor species based on previous phylogenetic study (Hilu and Alice 2001). They were used for constructing bootstrapped maximum-likelihood and neighbor-joining trees using MEGA X (Kumar et al. 2018) and Bayesian inference tree with MrBayes v3.2.7a (Ronquist et al. 2012) after aligning whole chloroplast genomes using MAFFT v7.450 (Katoh and Standley 2013). Phylogenetic trees show that our chloroplast genome is clustered as one clade with previously sequenced chloroplast genome of *Z. matrella* and *Zoysia tenuifolia* with high supportive values (Figure 1). Interestingly, only 4 SNPs and 19 INDELs were identified between our *Z. matrella* and *Z. tenuifolia*, which is smaller than intraspecific variations of *Z. matrella*. This phenomenon was also found in *Viburnum* (Choi YG et al. 2019) and *Pseudostellaria* (Kim, Heo, et al. 2019), displaying discrepancy between morphology and molecular markers. Because *Z. tenuifolia* is considered as *Zoysia pacifica* (Goudswaard 1980; Loch et al. 2017) and there are hybrids between *Z. tenuifolia* and *Z. pacifica* (Tanaka, Tokunaga, et al. 2016), this low number of variations between the two species require more investigation of the two species in both morphological and genetical aspects.

**Figure 1.**
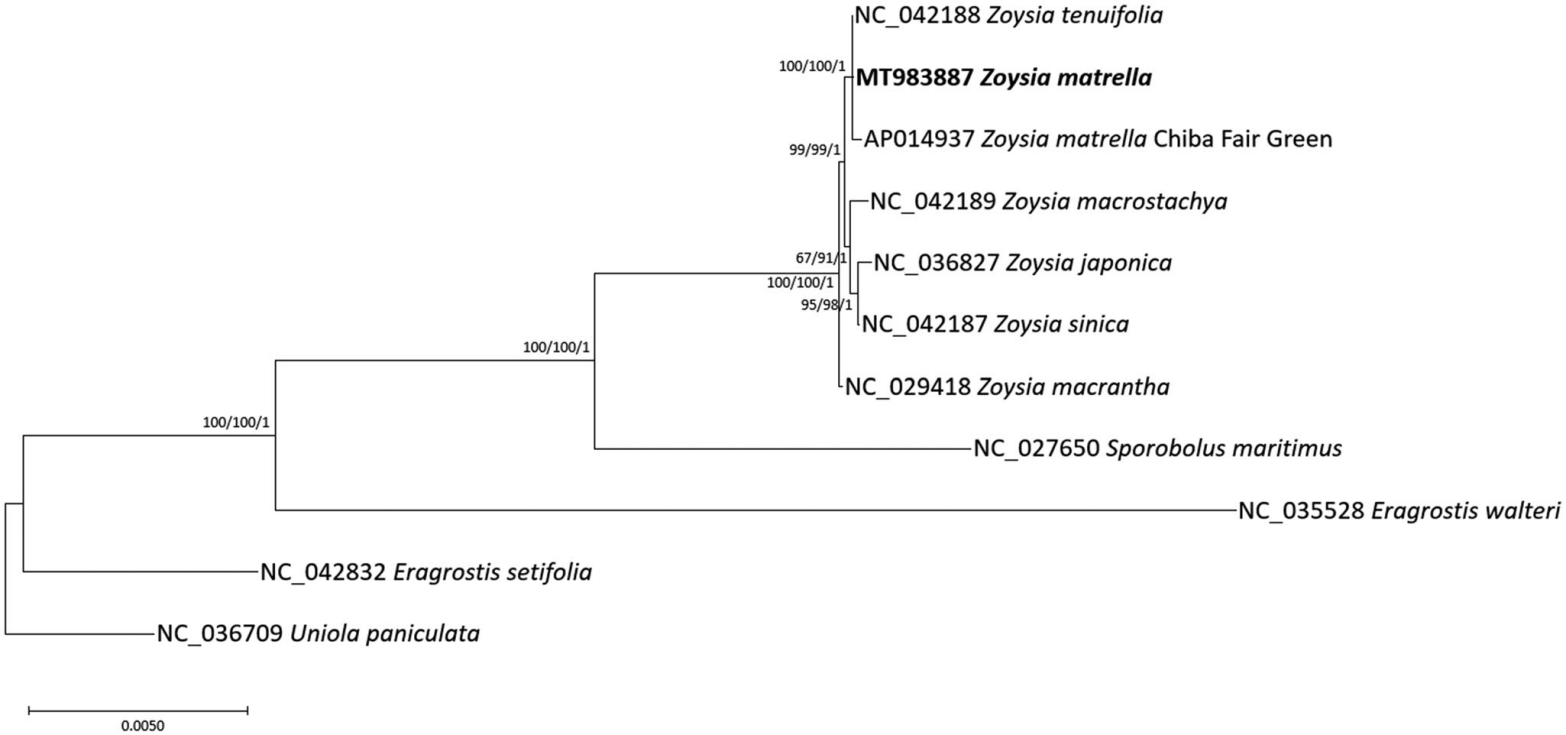
Phylogenetic tree was drawn based on maximum-likelihood tree based on alignment of eleven chloroplast genomes of *Zoysia* and neighbor species including one outgroup species. The numbers above branches indicate bootstrap support values of maximum likelihood, neighbor-joining, and Bayesian phylogenetic trees, respectively.

## Data Availability

Mitochondrial genome sequence can be accessed via accession number MT983887 in GenBank of NCBI at https://www.ncbi.nlm.nih.gov. The associated BioProject, SRA, and Bio-Sample numbers are PRJNA685656, SAMN17088999, and SRR13258751, respectively.
